# Cardioprotective Effects of **ω**-3 PUFAs in Chronic Kidney Disease

**DOI:** 10.1155/2013/712949

**Published:** 2013-04-04

**Authors:** Su Mi Lee, Won Suk An

**Affiliations:** ^1^Department of Internal Medicine, Seoul National University Hospital, 101 Daehak-Ro, Jongno-Gu, Seoul 110-744, Republic of Korea; ^2^Department of Internal Medicine, Dong-A University, 3Ga-1 Dongdaesin-Dong, Seo-Gu, Busan 602-715, Republic of Korea

## Abstract

The prevalence rate of chronic kidney disease (CKD) is increasing worldwide, and cardiovascular disease (CVD) is a main cause of death in patients with CKD. The high incidence of CVD in CKD patients is related to chronic inflammation, dyslipidemia, malnutrition, atherosclerosis, and vascular calcification. Omega-3 polyunsaturated fatty acids (**ω**-3 PUFAs) have been shown to reduce the risk of CVD. In this paper, we review the beneficial effects of **ω**-3 PUFAs on CVD and the possible cardioprotective mechanisms of **ω**-3 PUFAs in CKD patients by determining the effect of **ω**-3 PUFAs in the general population. **ω**-3 PUFAs have several cardioprotective benefits, such as reducing inflammation, decreasing oxidative stress, inhibiting platelet activity, exerting antiarrhythmic effects, and improving triglyceride levels, in the general population and patients with CKD. Modifications of erythrocyte membrane fatty acid content, including an increased **ω**-3 index and decreased oleic acid, after **ω**-3 PUFAs supplementation are important changes related to CVD risk reduction in the general population and patients with CKD. Further basic and clinical studies are essential to confirm the effects of **ω**-3 PUFAs on vitamin D activation, vascular calcification prevention, cardiovascular events, and mortality in CKD patients.

## 1. Introduction

Chronic kidney disease (CKD) is a public health problem, and the prevalence rate is increasing worldwide. The increasing prevalence rate of CKD is related to increased average life expectancy, the elderly population, obesity, diabetes, and hypertension, which are risk factors of CKD. Cardiovascular disease (CVD) is a main cause of death, the primary comorbid disease, and a frequent cause of hospitalization in patients with CKD [1, 2]. The high incidence of CVD in CKD patients is related to chronic inflammation, dyslipidemia, malnutrition, atherosclerosis, and vascular calcification [3–7]. Vascular calcification (VC) has been shown to be an independent predictor of cardiovascular mortality in CKD patients maintained on dialysis therapy. Therefore, preventive strategies for CVD are essential, especially in CKD patients.

Omega-3 polyunsaturated fatty acids (*ω*-3 PUFAs) supplementation has been linked to reducing the risk of CVD [8]. Dyerberg et al. found that Greenland Inuit who had a diet including a high content of *ω*-3 PUFAs had low mortality from coronary heart disease [9]. Subsequent studies found that *ω*-3 PUFAs intake is associated with a reduced risk of CVD [10]. This cardioprotective effect of *ω*-3 PUFAs is explained by its ability to suppress inflammation, inhibit platelet activation/adhesion, and reduce thrombosis [11]. The main effect of *ω*-3 PUFAs is reducing triglyceride levels in patients with hypertriglyceridemia, which is associated with CVD [12]. In addition, *ω*-3 PUFAs reduced oxidative stress and had the possibility to inhibit vascular calcification in human studies and a rat model [13–15]. Furthermore, *ω*-3 PUFAs may be involved in CVD by modulating cell membrane receptors and affecting signal transduction and eicosanoid metabolism [16–18]. Therefore, *ω*-3 PUFAs, which have several benefits in CVD, may be helpful to reduce CVD in CKD patients, who have a high prevalence rate of CVD. 

In this paper, we review the beneficial effects of *ω*-3 PUFAs on CVD and possible cardioprotective mechanisms of *ω*-3 PUFAs in CKD patients by evaluating the effect of *ω*-3 PUFAs in the general population. We also suggest several investigations to prove the cardioprotective effect of *ω*-3 PUFAs based on small challenging studies.

## 2. *ω*-3 PUFAs, *ω*-6 PUFAs, and the *ω*-3 Index

**Figure 1:**
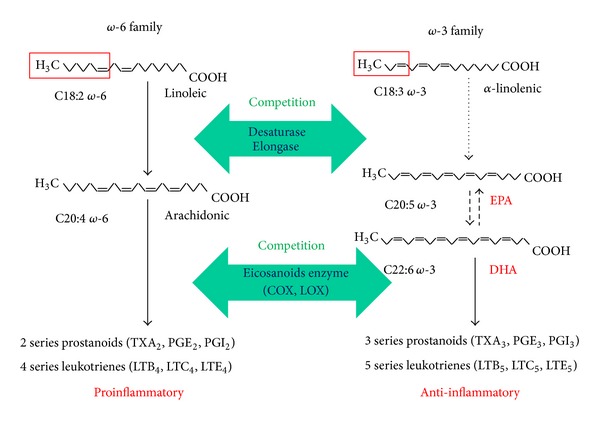
Biochemical pathways of *ω*-6 and *ω*-3 fatty acids.

*ω*-3 PUFAs are commonly found in marine and some plant oils, such as fish oils, algal oil, squid oil, echium oil, and flaxseed oil. They have several double bonds (C=C) beginning after the third carbon atom from the end of the carbon chain. They are considered as essential fatty acids, which cannot be synthesized by mammalian cells de novo but are vital for normal metabolism. *ω*-3 PUFAs include *α*-linolenic acid (ALA), eicosapentaenoic acid (EPA), docosapentaenoic acid (DPA), and docosahexaenoic acid (DHA) (Figure 1). ALA is a short-chain *ω*-3 PUFAs and can be converted into EPA and DHA, which are long-chain *ω*-3 PUFAs, by desaturase and elongase [19]. To support optimal EPA and DHA levels, these conversions are a rate-limited step and depend on the amount of dietary linoleic acid (LA) and ALA [19, 20]. 

Omega 6 PUFAs (*ω*-6 PUFAs) are another family of unsaturated  fatty acids that have a final carbon–carbon double bonds in the sixth bond counting from the methyl end. The major source of *ω*-6 PUFAs is animal meat. *ω*-6 PUFAs can be converted into arachidonic acid (AA) [21], and AA can be metabolized into *ω*-6 eicosanoid products, such as prostaglandins, leukotrienes, and thromboxanes, by cyclooxygenase, lipoxygenase, or cytochrome P450 AA monooxygenase. Excessive *ω*-6 PUFAs promote prothrombotic, proinflammatory, and atherosclerotic processes. Competitive interactions with *ω*-3 PUFAs affect the relative storage, mobilization, conversion, and action of the *ω*-3 and *ω*-6 eicosanoid precursors.

Harris and Von Schacky announced the omega-3 index (*ω*-3 index), a new risk factor for death caused by coronary artery disease (CAD) [22]. This index is defined as the percentage of EPA + DHA content in red blood cells (RBCs) membranes. The rationale for this index is that the FA content of erythrocyte membranes has been shown to highly correlate with the FA content of the myocardium [23]. The erythrocyte FA composition is less variable than plasma. Therefore, the cardiac FA content can be determined by estimating the erythrocyte membrane FA content. The modification of the FA content can either reduce or increase the risks of cardiovascular events. It is necessary to measure the erythrocyte membrane FA content, including the *ω*-3 index, which can be affected by dietary habits, especially in clinical trials for evaluating the effect of *ω*-3 PUFAs.

## 3. Anti-Inflammatory and Antithrombotic Effects of *ω*-3 PUFAs

Inflammation results from immunological processes in response to injury, invading pathogens, allergens, and toxins and leads to the repair of damaged tissue. Chronic and persistent inflammation plays a central role in the development and progression of CAD. The inflammatory response is regulated by a complex network of mediators, including lipid mediators (eicosanoids, docosanoids, and platelet-activating factors), cytokines, and chemokines [24]. These mediators promote platelet aggregation and have proinflammatory effects. 

*ω*-6 PUFAs are converted to AA-derived eicosanoids, which have prothrombotic, proinflammatory, and proarteriosclerotic effects. In contrast, *ω*-3 PUFAs compete with the AA cascade, and *ω*-3 PUFAs consumption increases EPA, *ω*-3 PUFAs in the cell membrane. EPA is a competitive inhibitor of cyclooxygenase. EPA reduces the production of the 2-series prostaglandins, thromboxanes, and prostacyclins and the 4-series leukotrienes and produces the 3- and 5-series prostanoids, which are less biologically active. DHA can inhibit AA metabolism and platelet aggregation by reducing the affinity of the platelet TXA2/PGH2 receptor. These actions of *ω*-3 PUFAs may contribute to benefits regarding CVD. *ω*-3 PUFAs also decrease the risk of thrombosis by inhibiting platelet aggregation [25]. The effects of *ω*-3 PUFAs on platelet function and thrombosis are still controversial. Some studies have reported that there is no significant association between *ω*-3 PUFAs supplementation and coagulation factors [26]. Therefore, further investigations on *ω*-3 PUFAs are necessary to clarify its antithrombotic effect.

## 4. Anti-Inflammatory and Antithrombotic Effects of *ω*-3 PUFAs in CKD

CKD is a microinflammatory state, and inflammation is a salient feature in CKD patients on predialysis and dialysis [27]. Previous studies have shown that renal dysfunction is associated with inflammation. In a study using data from NHANES III, C-reactive protein (CRP), serum homocysteine, and plasma fibrinogen levels were elevated in CKD patients [28]. These inflammatory markers may play a role in increasing the cardiovascular risk among patients with CKD. Increased inflammatory mediators have been associated with increased oxidative stress and the accumulation of advanced glycation end (AGE) products. Various studies have reported that omega-3 fatty acid (*ω*-3 FA) can decrease inflammatory markers [29–31]. *ω*-3 PUFAs supplementation significantly attenuated oxidative and inflammatory pathways by decreasing NOX-4, gp91phox, p47phox, p22phox, MCP-1, NF kappa-B, and COX-2 expression in a 5/6 nephrectomy rat model [32]. As a result of *ω*-3 PUFAs actions, renal fibrosis was also decreased [32]. Recent studies have reported that resolvins and protectins, which are a new class of lipid mediators, are associated with the resolution of renal inflammation [33]. *ω*-3 PUFAs are used as an additional treatment for inflammatory diseases, such as rheumatoid arthritis [34]. However, some studies have reported that *ω*-3 PUFAs intake does not have anti-inflammatory benefits in CKD patients [35]. Further clinical studies are required to identify the anti-inflammatory effect of *ω*-3 PUFAs in CKD patients with chronic inflammation. 

## 5. Antiarrhythmic Effects of *ω*-3 PUFAs

*ω*-3 PUFAs have beneficial effects on cardiovascular health and mortality through reducing arrhythmia in the myocardium. *ω*-3 PUFAs may contribute to reducing the resting heart rate, promoting a faster return to resting heart rate after exercise, and increasing heart rate variability and left ventricular filling capacity [36–38]. The mechanism of these benefits is derived from preventing calcium overload by maintaining the activity of L-type calcium channels during periods of stress and increasing the activity of cardiac microsomal Ca^2+^/Mg^2+^ ATPase (adenosine triphosphatase). In addition, *ω*-3 PUFAs may attenuate delayed depolarization by reducing Na^+^/Ca^2+^ exchange currents, which may alter membrane electrical activity [39, 40]. Thus, *ω*-3 PUFAs increase myocardiocyte membrane electrical stability and thereby prevent malignant dysrhythmia [17]. However, further basic studies are necessary to elucidate how *ω*-3 PUFAs stabilize these channels and are involved in membrane activity. Still, there are few studies on the effect of *ω*-3 PUFAs on arrhythmia in CKD patients, and further clinical studies are necessary.

## 6. Effects of *ω*-3 PUFAs on Erythrocyte Membrane Fatty Acid Content in CKD

Fatty acids are required for membrane synthesis and protein and carbohydrate modification, and the necessity of specific fatty acid compositions is different according to specific medical conditions. In several studies, *ω*-3 PUFAs supplementation augmented the erythrocyte membrane EPA and DHA content and consequently the *ω*-3 index, in CVD patients. These modifications of erythrocyte membrane fatty acid content were also shown in CKD patients. *ω*-3 PUFAs supplementation may be helpful for reducing the risk of CVD with regard to the increased content of *ω*-3 PUFAs, and consequently the *ω*-3 index. However, the precise mechanism underlying the cardioprotective effect and how increased amounts of *ω*-3 PUFAs affect the cellular functions and cell membrane receptors are unknown. Further studies are needed to evaluate the effect of *ω*-3 PUFAs on the conformational changes of membrane receptors and functional changes of cell membrane receptors. 

A higher intake of saturated fatty acids increases the cell membrane content of total saturated fatty acid and is related to an increased incidence of CAD [41, 42]. Therefore, decreasing erythrocyte total saturated fatty acid content may be helpful to reduce the incidence of CAD. The total saturated fatty acid content was decreased by *ω*-3 PUFAs supplementation in the general population and CKD patients [43–45].

Oleic acid has been shown to stimulate vascular smooth muscle cell migration and proliferation via the direct activation of extracellular signal-regulated kinase *in vitro*. In addition, oleic acid amplifies angiotensin II-induced protein kinase C (PKC) activation and reactive oxygen species generation *in vitro* [46, 47]. Erythrocyte membrane oleic acid levels were significantly higher in patients with acute coronary syndrome compared with a control group [48, 49]. In a previous study, erythrocyte membrane oleic acid levels were also elevated in CKD patients, who are at higher risk for CVD, treated with dialysis [11, 43]. Furthermore, erythrocyte membrane oleic acid levels were associated with the vascular calcification score on plain radiographs, which was related to CVD and CAD in hemodialysis patients [50]. Therefore, based on these results, erythrocyte membrane oleic acid levels may be related to CVD. *ω*-3 PUFAs supplementation decreased erythrocyte membrane oleic acid levels in CKD patients treated with dialysis [43, 44]. One study showed that a 30% reduction in arachidonic acid/EPA ratio was associated with a 70% reduction in the risk of myocardial infarction [31]. The AA/EPA ratio can be easily reduced by *ω*-3 PUFAs because *ω*-3 PUFAs can decrease erythrocyte membrane content of AA, which is related to inflammatory process. The modification of fatty acid content in erythrocyte membranes by *ω*-3 PUFAs plays an important role in preventing CVD. Several studies have reported an inverse relationship between renal function and risk of CVD [2, 4]. Further investigations may be needed to evaluate the modification of erythrocyte membrane fatty acid content according to renal function in CKD patients. 

## 7. Effects of *ω*-3 PUFAs on Lipid Metabolism in CKD

Another main effect of *ω*-3 PUFAs is regulating lipid homeostasis. *ω*-3 PUFAs affect lipid metabolism by decreasing the synthesis of very low-density lipoprotein (VLDL), promoting *β*-oxidation in mitochondria and/or peroxisomes, and reducing remnant lipoprotein levels, such as apo-B degradation [51–53]. *ω*-3 PUFAs especially decrease triglyceride levels by inhibiting the activities of diacylglycerol acyltransferase and phosphatidic acid phosphohydrolase. Recently, high triglyceride levels and low high-density lipoprotein (HDL) cholesterol were identified as residual risk factors for CVD in patients with strictly controlled low-density lipoprotein (LDL) cholesterol [54]. Therefore, physicians occasionally prescribe *ω*-3 PUFAs to control hypertriglyceridemia.

The primary finding of dyslipidemia in CKD and dialysis patients is hypertriglyceridemia. Over 40 percent of patients with CKD have triglyceride levels greater than 200 mg/dL. Therefore, *ω*-3 PUFAs are useful for controlling dyslipidemia and reducing CVD risk in CKD patients. However, the total cholesterol concentration, triglyceride level, and LDL cholesterol are sometimes normal or low in CKD patients with malnutrition [55]. In fact, *ω*-3 PUFAs supplementation did not affect triglyceride levels in dialysis patients with normal triglyceride levels [43, 44].

## 8. Vitamin D and *ω*-3 PUFAs in CKD 

Vitamin D deficiency is a common condition that affects one billion people worldwide, and the prevalence rate of this condition is increasing [56]. Vitamin D deficiency leads to many health problems, such as CVD, hypertension, insulin resistance, diabetes mellitus, and cancer progression [57–60]. Although there is evidence of vitamin D deficiency affecting CVD, the mechanisms underlying how vitamin D protects the cardiovascular system are unclear. *In vitro* and clinical studies suggest that vitamin D receptor activation leads to downregulation of the renin-angiotensin system, inflammation inhibition, smooth muscle proliferation suppression, and vascular calcification [61–63]. Vitamin D receptor knockout mice develop hypertension and cardiac hypertrophy [64]. Epidemiologic studies have reported that vitamin D deficiency is associated with cardiovascular events in subjects with renal dysfunction and even in the general population [65, 66]. Vitamin D deficiency is much more common in patients with decreased renal function than in those with normal renal function. Several studies have reported an association between vitamin D deficiency and CVD in CKD patients [67, 68]. Thus, providing proper vitamin D supplementation may contribute to public health benefits similar to *ω*-3 PUFAs supplementation. The Vitamin D and Omega-3 Trial (VITAL), a randomized, double-blind, placebo-controlled, large-scale intervention trial, is currently ongoing. The VITAL study is evaluating whether vitamin D and *ω*-3 PUFAs reduce the risk of cancer and major cardiovascular events and is recruiting 20,000 participants who have no previous illness. The results of the VITAL study may define the effect of vitamin D and *ω*-3 PUFAs in the primary prevention of CVD.

Vitamin D is hydroxylated to 25(OH)D in the liver and is then converted to a potent biological metabolite (1,25(OH)2D) by the enzyme 1*α*-hydroxylase [69]. The biologically active metabolite 1,25(OH)2D has anti-inflammatory and antiproliferative effects on the endothelial cells of the vascular wall [70]. A recent study showed that 1,25(OH)2D concentrations were significantly increased after 3 and 6 months in a *ω*-3 PUFAs supplemented group compared to baseline in dialysis patients [44]. Therefore, further studies are needed to confirm the cardioprotective effect of *ω*-3 PUFAs through activating vitamin D.

## 9. Vascular Calcification and *ω*-3 PUFAs in CKD

Vascular calcification is highly prevalent in patients with CKD, and it is an independent predictor of cardiovascular mortality in CKD patients [71]. *ω*-3 PUFAs have a beneficial effect on the vascular system by reducing pulse wave velocity. The pulse wave velocity is associated with vascular calcification on plain radiographs in subjects on dialysis [72]. Fetuin-A also antagonizes the vascular calcifying effects of bone morphogenetic protein-2 [73]. A recent study showed that fetuin-A levels after *ω*-3 PUFAs supplementation were significantly increased in dialysis patients. However, whether vascular calcification is inhibited by *ω*-3 PUFAs is unknown, despite an animal study [15]. Further prospective studies are necessary to evaluate the effects of *ω*-3 PUFAs on preventing vascular calcification in CKD patients.

## 10. Effects of *ω*-3 PUFAs on Cardiovascular Events and Mortality in CKD

Several clinical trials have reported that elevated *ω*-3 PUFAs levels reduce the risk of CVD. The Diet and Reinfarction Trial (DART) investigated the effect of dietary intervention in patients with recent myocardial infarction [74]. The patients in the fatty fish advice group showed decreased mortality. In the Gruppo Ialiano per la Sperimentazione della Streptochinasi nell'infarto Miocardio Prevenzione (GISSI) trial, the *ω*-3 PUFAs supplemented group demonstrated a reduction in cardiovascular death, coronary death, and sudden cardiac death [8]. In 2002, the American Heart Association (AHA) recommended that subjects with heart disease ingest 1 g fish oil daily. The AHA also recommends that CKD patients who have a high risk of CVD consume at least 1 g of *ω*-3 PUFAs PO daily. However, some studies have reported that *ω*-3 PUFAs are not significantly associated with a cardioprotective effect. Kromhout et al. demonstrated that patients who had previous myocardial infarction and were undergoing proper medical care did not show a reduced rate of cardiovascular events despite supplementation with low-dose EPA-DHA [75]. Another randomized clinical trial reported that *ω*-3 PUFAs supplementation was not associated with cardiovascular events [76]. Some studies did not survey actual dietary habits, and some studies did not measure plasma or membrane *ω*-3 PUFAs levels, including the *ω*-3 index. These points are very important to interpret the results of clinical trials. In addition, the doses of supplemented *ω*-3 PUFAs are another important point affecting the results of clinical trials. To our knowledge, there are no large-scale clinical trials with inclusion criteria based on the baseline content of *ω*-3 PUFAs. Therefore, the effects of *ω*-3 PUFAs supplementation may be different according to the doses and baseline content of *ω*-3 PUFAs. In addition, currently, many patients and healthy individuals frequently consume healthy food and pills derived from healthy food, such as *ω*-3 PUFAs and multivitamins. Patients with CVD are highly informed regarding cardiovascular health and take cardioprotective drugs, such as angiotensin-converting enzyme inhibitors or statins. These environmental factors affect the results of clinical trials on CVD and CVD-related mortality. In CKD patients, more factors are related to CVD and CVD-related mortality. *ω*-3 PUFAs studies have identified various clinical outcomes that are relevant to patients with renal dysfunction. *ω*-3 PUFAs supplementation may reduce the cardiovascular risk in subjects with decreased renal function and even in dialysis patients [77]. Another study has shown that *ω*-3 PUFAs may have beneficial effects against CVD through improving blood pressure and heart rate [78]. Nevertheless, the cardioprotective effect of *ω*-3 PUFAs is still controversial. Larger controlled clinical trials are needed to establish the cardioprotective effect of *ω*-3 PUFAs in CKD patients.

## 11. Conclusions

**Figure 2:**
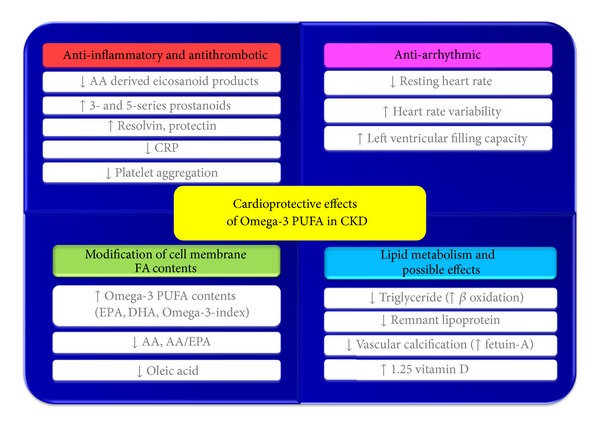
Cardioprotective effects of *ω*-3 PUFAs in chronic kidney disease (CKD).

*ω*-3 PUFAs have several benefits for minimizing CVD risks by reducing inflammation, decreasing oxidative stress, inhibiting platelet activity, exerting antiarrhythmic effects, and improving triglyceride levels in the general population and patients with CKD (Figure 2). In addition, the modification of erythrocyte membrane fatty acid content by *ω*-3 PUFAs supplementation is an important process related to CVD risk reduction which may help in the interpretation of clinical trials in general populations and patients with CKD. Increasing erythrocyte membrane content of *ω*-3 PUFAs, and consequently the *ω*-3 index, and decreasing total saturated fatty acids, oleic acids, and AA may affect cellular function by changing transmembrane proteins and inflammatory mediators involved with cell signaling systems. The role of *ω*-3 PUFAs in vitamin D activation, vascular calcification prevention, cardiovascular events, and mortality should be further investigated in CKD patients. 

## References

[1] Foley RN, Parfrey PS, Sarnak MJ (1998). Clinical epidemiology of cardiovascular disease in chronic renal disease. *American Journal of Kidney Diseases*.

[2] Go AS, Chertow GM, Fan D, McCulloch CE, Hsu CY (2004). Chronic kidney disease and the risks of death, cardiovascular events, and hospitalization. *The New England Journal of Medicine*.

[3] Stenvinkel P, Heimbürger O, Lindholm B, Kaysen GA, Bergström J (2000). Are there two types of malnutrition in chronic renal failure? Evidence for relationships between malnutrition, inflammation and atherosclerosis (MIA syndrome). *Nephrology Dialysis Transplantation*.

[4] Sarnak MJ, Levey AS, Schoolwerth AC (2003). Kidney disease as a risk factor for development of cardiovascular disease: a statement from the American Heart Association Councils on Kidney in Cardiovascular Disease, High Blood Pressure Research, Clinical Cardiology, and Epidemiology and Prevention. *Hypertension*.

[5] Schiffrin EL, Lipman ML, Mann JFE (2007). Chronic kidney disease: effects on the cardiovascular system. *Circulation*.

[6] Harper CR, Jacobson TA (2008). Managing dyslipidemia in chronic kidney disease. *Journal of the American College of Cardiology*.

[7] Mizobuchi M, Towler D, Slatopolsky E (2009). Vascular calcification: the killer of patients with chronic kidney disease. *Journal of the American Society of Nephrology*.

[8] Marchioli R (1999). Dietary supplementation with N-3 polyunsaturated fatty acids and vitamin E after myocardial infarction: results of the GISSI-Prevenzione trial. *The Lancet*.

[9] Dyerberg J, Bang HO, Hjorne N (1975). Fatty acid composition of the plasma lipids in Greenland Eskimos. *American Journal of Clinical Nutrition*.

[10] Hu FB, Bronner L, Willett WC (2002). Fish and omega-3 fatty acid intake and risk of coronary heart disease in women. *Journal of the American Medical Association*.

[11] An WS, Kim SE, Kim KH (2009). Comparison of fatty acid contents of erythrocyte membrane in hemodialysis and peritoneal dialysis patients. *Journal of Renal Nutrition*.

[12] Eslick GD, Howe PRC, Smith C, Priest R, Bensoussan A (2009). Benefits of fish oil supplementation in hyperlipidemia: a systematic review and meta-analysis. *International Journal of Cardiology*.

[13] Tomiyama H, Takazawa K, Osa SI (2005). Do eicosapentaenoic acid supplements attenuate age-related increases in arterial stiffness in patients with dyslipidemia?: a preliminary study. *Hypertension Research*.

[14] Hall WL, Sanders KA, Sanders TAB, Chowienczyk PJ (2008). A high-fat meal enriched with eicosapentaenoic acid reduces postprandial arterial stiffness measured by digital volume pulse analysis in healthy men. *Journal of Nutrition*.

[15] Kanai S, Uto K, Honda K, Hagiwara N, Oda H (2011). Eicosapentaenoic acid reduces warfarin-induced arterial calcification in rats. *Atherosclerosis*.

[16] Kang JX, Leaf A (1996). Evidence that free polyunsaturated fatty acids modify Na+ channels by directly binding to the channel proteins. *Proceedings of the National Academy of Sciences of the United States of America*.

[17] Leaf A (2001). The electrophysiologic basis for the antiarrhythmic and anticonvulsant effects of n-3 polyunsaturated fatty acids: heart and brain. *Lipids*.

[18] Shearer GC, Pottala JV, Spertus JA, Harris WS (2009). Red blood cell fatty acid patterns and acute coronary syndrome. *PLoS ONE*.

[19] Hussein N, Ah-Sing E, Wilkinson P, Leach C, Griffin BA, Millward DJ (2005). Long-chain conversion of [13C]linoleic acid and *α*-linolenic acid in response to marked changes in their dietary intake in men. *Journal of Lipid Research*.

[20] Friedman A, Moe S (2006). Review of the effects of omega-3 supplementation in dialysis patients. *Clinical Journal of the American Society of Nephrology*.

[21] Grimsgaard S, Bønaa KH, Hansen JB, Myhre ESP (1998). Effects of highly purified eicosapentaenoic acid and docosahexaenoic acid on hemodynamics in humans. *American Journal of Clinical Nutrition*.

[22] Harris WS, Von Schacky C (2004). The Omega-3 Index: a new risk factor for death from coronary heart disease?. *Preventive Medicine*.

[23] Harris WS, Sands SA, Windsor SL (2004). Omega-3 fatty acids in cardiac biopsies from heart transplantation patients: correlation with erythrocytes and response to supplementation. *Circulation*.

[24] Rangel-Huerta OD, Aguilera CM, Mesa MD, Gil A (2012). Omega-3 long-chain polyunsaturated fatty acids supplementation on inflammatory biomakers: a systematic review of randomised clinical trials. *British Journal of Nutrition*.

[25] Ågren JJ, Väisänen S, Hänninen O, Muller AD, Hornstra G (1997). Hemostatic factors and platelet aggregation after a fish-enriched diet or fish oil or docosahexaenoic acid supplementation. *Prostaglandins Leukotrienes and Essential Fatty Acids*.

[26] Archer SL, Green D, Chamberlain M, Dyer AR, Liu K (1998). Association of dietary fish and n-3 fatty acid intake with hemostatic factors in the coronary artery risk development in young adults (CARDIA)study. *Arteriosclerosis, Thrombosis, and Vascular Biology*.

[27] Kaysen GA (2001). The microinflammatory state in uremia: causes and potential consequences. *Journal of the American Society of Nephrology*.

[28] Muntner P, Hamm LL, Kusek JW, Chen J, Whelton PK, He J (2004). The prevalence of nontraditional risk factors for coronary heart disease in patients with chronic kidney disease. *Annals of Internal Medicine*.

[29] Pischon T, Hankinson SE, Hotamisligil GS, Rifai N, Willett WC, Rimm EB (2003). Habitual dietary intake of n-3 and n-6 fatty acids in relation to inflammatory markers among US men and women. *Circulation*.

[30] Perunicic-Pekovic GB, Rasic ZR, Pljesa SI (2007). Effect of n-3 fatty acids on nutritional status and inflammatory markers in haemodialysis patients. *Nephrology*.

[31] Hassan KS, Hassan SK, Hijazi EG, Khazim KO (2010). Effects of omega-3 on lipid profile and inflammation markers in peritoneal dialysis patients. *Renal Failure*.

[32] An WS, Kim HJ, Cho KH, Vaziri ND (2009). Omega-3 fatty acid supplementation attenuates oxidative stress, inflammation, and tubulointerstitial fibrosis in the remnant kidney. *American Journal of Physiology*.

[33] Serhan CN, Chiang N, Van Dyke TE (2008). Resolving inflammation: dual anti-inflammatory and pro-resolution lipid mediators. *Nature Reviews Immunology*.

[34] James M, Proudman S, Cleland L (2010). Fish oil and rheumatoid arthritis: past, present and future. *Proceedings of the Nutrition Society*.

[35] Deike E, Bowden RG, Moreillon JJ (2012). The effects of fish oil supplementation on markers of inflammation in chronic kidney disease patients. *Journal of Renal Nutrition*.

[36] O’Keefe JH, Abuissa H, Sastre A, Steinhaus DM, Harris WS (2006). Effects of omega-3 fatty acids on resting heart rate, heart rate recovery after exercise, and heart rate variability in men with healed myocardial infarctions and depressed ejection fractions. *American Journal of Cardiology*.

[37] Mozaffarian D, Prineas RJ, Stein PK, Siscovick DS (2006). Dietary fish and n-3 fatty acid intake and cardiac electrocardiographic parameters in humans. *Journal of the American College of Cardiology*.

[38] Kang JX (2012). Reduction of heart rate by omega-3 fatty acids and the potential underlying mechanisms. *Frontiers in Cardiac Electrophysiology*.

[39] Kris-Etherton PM, Harris WS, Appel LJ (2002). Fish consumption, fish oil, omega-3 fatty acids, and cardiovascular disease. *Circulation*.

[40] Zhao YT, Chen Q, Sun YX (2009). Prevention of sudden cardiac death with omega-3 fatty acids in patients with coronary heart disease: a meta-analysis of randomized controlled trials. *Annals of Medicine*.

[41] Katan MB, Zock PL, Mensink RP (1995). Dietary oils, serum lipoproteins, and coronary heart disease. *American Journal of Clinical Nutrition*.

[42] Grundy SM, Hegsted DM, Astrup A, Hill JO, Saris WHM, Taubes G (2001). Dietary fat: at the heart of the matter. *Science*.

[43] An WS, Lee SM, Son YK (2012). Effect of omega-3 fatty acids on the modification of erythrocyte membrane fatty acid content including oleic acid in peritoneal dialysis patients. *Prostaglandins Leukotrienes and Essential Fatty Acids*.

[44] An WS, Lee SM, Son YK (2012). Omega-3 fatty acid supplementation increases 1, 25-dihydroxyvitamin D and fetuin-A levels in dialysis patients. *Nutrition Research*.

[45] Riediger ND, Othman RA, Suh M, Moghadasian MH (2009). A systemic review of the roles of n-3 fatty acids in health and disease. *Journal of the American Dietetic Association*.

[46] Egan BM, Lu G, Greene EL (1999). Vascular effects of non-esterified fatty acids: implications for the cardiovascular risk factor cluster. *Prostaglandins Leukotrienes and Essential Fatty Acids*.

[47] Lu G, Greene EL, Nagai T, Egan BM (1998). Reactive oxygen species are critical in the oleic acid-mediated mitogenic signaling pathway in vascular smooth muscle cells. *Hypertension*.

[48] Block RC, Harris WS, Reid KJ, Spertus JA (2008). Omega-6 and trans fatty acids in blood cell membranes: a risk factor for acute coronary syndromes?. *American Heart Journal*.

[49] Paganelli F, Maixent JM, Duran MJ, Parhizgar R, Pieroni G, Sennoune S (2001). Altered erythrocyte n-3 fatty acids in Mediterranean patients with coronary artery disease. *International Journal of Cardiology*.

[50] Son YK, Lee SM, Kim SE (2012). Association between vascular calcification scores on plain radiographs and fatty acid contents of erythrocyte membrane in hemodialysis patients. *Journal of Renal Nutrition*.

[51] Ando M, Sanaka T, Nihei H (1999). Eicosapentanoic acid reduces plasma levels of remnant lipoproteins and prevents in vivo peroxidation of LDL in dialysis patients. *Journal of the American Society of Nephrology*.

[52] Sampath H, Ntambi JM (2005). Polyunsaturated fatty acid regulation of genes of lipid metabolism. *Annual Review of Nutrition*.

[53] Harris WS, Bulchandani D (2006). Why do omega-3 fatty acids lower serum triglycerides?. *Current Opinion in Lipidology*.

[54] Kearney PM, Blackwell L, Collins R (2008). Efficacy of cholesterol-lowering therapy in 18 686 people with diabetes in 14 randomised trials of statins: a meta-analysis. *The Lancet*.

[55] Weiner DE, Sarnak MJ (2004). Managing dyslipidemia in chronic kidney disease. *Journal of General Internal Medicine*.

[56] Holick MF (2007). Medical progress: vitamin D deficiency. *The New England Journal of Medicine*.

[57] Garland CF, Gorham ED, Mohr SB, Garland FC (2009). Vitamin D for cancer prevention: global perspective. *Annals of Epidemiology*.

[58] Mezawa H, Sugiura T, Watanabe M (2010). Serum vitamin D levels and survival of patients with colorectal cancer: post-hoc analysis of a prospective cohort study. *BMC Cancer*.

[59] Danescu LG, Levy S, Levy J (2009). Vitamin D and diabetes mellitus. *Endocrine*.

[60] Kayaniyil S, Vieth R, Retnakaran R (2010). Association of vitamin D with insulin resistance and *β*-cell dysfunction in subjects at risk for type 2 diabetes. *Diabetes Care*.

[61] Theuer J, Shagdarsuren E, Muller DN (2005). Inducible NOS inhibition, eicosapentaenoic acid supplementation, and angiotensin II-induced renal damage. *Kidney International*.

[62] Rigby WFC, Denome S, Fanger MW (1987). Regulation of lymphokine production and human T lymphocyte activation by 1,25-dihydroxyvitamin D3. Specific inhibition at the level of messenger RNA. *The Journal of Clinical Investigation*.

[63] Li YC, Qiao G, Uskokovic M, Xiang W, Zheng W, Kong J (2004). Vitamin D: a negative endocrine regulator of the renin-angiotensin system and blood pressure. *Journal of Steroid Biochemistry and Molecular Biology*.

[64] Li YC, Kong J, Wei M, Chen ZF, Liu SQ, Cao LP (2002). 1,25-Dihydroxyvitamin D3 is a negative endocrine regulator of the renin-angiotensin system. *The Journal of Clinical Investigation*.

[65] Wang TJ, Pencina MJ, Booth SL (2008). Vitamin D deficiency and risk of cardiovascular disease. *Circulation*.

[66] Melamed ML, Michos ED, Post W, Astor B (2008). 25-hydroxyvitamin D levels and the risk of mortality in the general population. *Archives of Internal Medicine*.

[67] Melamed ML, Astor B, Michos ED, Hostetter TH, Powe NR, Muntner P (2009). 25-Hydroxyvitamin D levels, race, and the progression of kidney disease. *Journal of the American Society of Nephrology*.

[68] Santoro D, Gitto L, Ferraro A, Satta E, Savica V, Bellinghieri G (2011). Vitamin D status and mortality risk in patients with chronic kidney disease. *Renal Failure*.

[69] Zehnder D, Bland R, Walker EA (1999). Expression of 25-hydroxyvitamin D3-1*α*-hydroxylase in the human kidney. *Journal of the American Society of Nephrology*.

[70] Artaza JN, Mehrotra R, Norris KC (2009). Vitamin D and the cardiovascular system. *Clinical Journal of the American Society of Nephrology*.

[71] Shobeiri N PJ, Adams MA, Holden RM (2013). Cardiovascular disease in an adenine-induced model of chronic kidney disease: the temporal link between vascular calcification and haemodynamic consequences. *Journal of Hypertension*.

[72] Nishizawa H, Hamazaki K, Hamazaki T, Fujioka S, Sawazaki S (2006). The relationship between tissue RBC n-3 fatty acids and pulse wave velocity. *In Vivo*.

[73] Szweras M, Liu D, Partridge EA (2002). *α*2-HS glycoprotein/fetuin, a transforming growth factor *β*/bone morphogenetic protein antagonist, regulates postnatal bone growth and remodeling. *The Journal of Biological Chemistry*.

[74] Burr ML, Fehily AM, Gilbert JF (1989). Effects of changes in fat, fish, and fibre intakes on death and myocardial reinfarction: diet and reinfarction trial (DART). *The Lancet*.

[75] Kromhout D, Giltay EJ, Geleijnse JM (2010). N-3 fatty acids and cardiovascular events after myocardial infarction. *The New England Journal of Medicine*.

[76] Rizos EC, Ntzani EE, Bika E, Kostapanos MS, Elisaf MS (2012). Association between omega-3 fatty acid supplementation and risk of major cardiovascular disease events: a systematic review and meta-analysis. *Journal of the American Medical Association*.

[77] Christensen JH, Aarøe J, Knudsen N (1998). Heart rate variability and n-3 fatty acids in patients with chronic renal failure—a pilot study. *Clinical Nephrology*.

[78] Mori TA, Burke V, Puddey IB (2009). The effects of *ω*3 fatty acids and coenzyme Q10 on blood pressure and heart rate in chronic kidney disease: a randomized controlled trial. *Journal of Hypertension*.

